# Keystone Veterinary Medical Association

**Published:** 1898-03

**Authors:** W. L. Rhoads

**Affiliations:** Secretary


Secretary.

### KEYSTONE VETERINARY MEDICAL ASSOCIATION.

The February meeting was held on the 8th inst., Dr. Thomas B. Rayner presiding, as both President and Vice-President were absent. But few of our members responded to roll-call. Those present were Drs. W. H. Hoskins, W. S. Kooker, W. L. Rhoads, T. B. Rayner, C. J. Marshall, and A. N. Lushington. Our list of visitors was also short at this meeting, but three or four being present. Among the visitors were Drs. J. N. Megary and H. D. Hackler.

After the regular routine of business and discussion of the resolution offered at previous meeting, to make the payment of five dollars initiation fee cover dues to succeeding annual meeting, it was unanimously adopted. Drs. Marshall and Houldsworth made a report at some length upon the extent to which dairies were under veterinary sanitary supervision. Dr. Hoskins made a report upon the agitation at present with the Board of Health regarding methods of serving milk. Dr. Rayner appointed Dr. Pearson on the committee already appointed to keep in touch with these meetings.

Dr. C. J. Marshall now read an interesting paper on the "Better Methods of Handling Milk." Notice was now given of the annual meeting of the Pennsylvania State Veterinary Medical Association, to be held in Philadelphia, March 8th, when the meeting adjourned to meet March 8th in conjunction with State Association.

DR. W. L. RHOADS,
Secretary.

